# Single-cell sequencing reveals the heterogeneity of B cells and tertiary lymphoid structures in muscle-invasive bladder cancer

**DOI:** 10.1186/s12967-024-04860-1

**Published:** 2024-01-12

**Authors:** Hao Yuan, Xingning Mao, Yunkun Yan, Rong Huang, Qingyun Zhang, Yanyu Zeng, Mengying Bao, Yan Dai, Bo Fang, Junhao Mi, Yuli Xie, Xiang Wang, Haiying Zhang, Zengnan Mo, Rirong Yang

**Affiliations:** 1 Center for Genomic and Personalized Medicine, Guangxi key Laboratory for Genomic and Personalized Medicine, Guangxi Collaborative Innovation Center for Genomic and Personalized Medicine, Guangxi Medical University, Nanning, 530021 Guangxi China; 2 Department of Immunology, School of Basic Medical Sciences, Guangxi Medical University, Nanning, 530021 Guangxi China; 3 Guangxi Collaborative Innovation Center for Biomedicine, Guangxi Medical University, Nanning, 530021 Guangxi China; 4 Collaborative Innovation Centre of Regenerative Medicine and Medical BioResource Development and Application Co-constructed By the Province and Ministry, Guangxi Medical University, Nanning, 530021 Guangxi China; 5 Department of Urology, the Affiliated Tumor Hospital of Guangxi Medical University, Guangxi Medical University, Nanning, 530021 Guangxi China; 6 Department of Occupational Health and Environmental Health, School of Public Health, Guangxi Medical University, Nanning, 530021 Guangxi China; 7 Institute of Urology and Nephrology, the First Affiliated Hospital of Guangxi Medical University, Guangxi Medical University, Nanning, 530021 Guangxi China

**Keywords:** B cells, Tertiary lymphoid structures, Muscle-invasive bladder cancer, Single-cell sequencing, CXCL13

## Abstract

**Background:**

Muscle-invasive bladder cancer (MIBC) is a highly aggressive disease with a poor prognosis. B cells are crucial factors in tumor suppression, and tertiary lymphoid structures (TLSs) facilitate immune cell recruitment to the tumor microenvironment (TME). However, the function and mechanisms of tumor-infiltrating B cells and TLSs in MIBC need to be explored further.

**Methods:**

We performed single-cell RNA sequencing analysis of 11,612 B cells and 55,392 T cells from 12 bladder cancer patients and found naïve B cells, proliferating B cells, plasma cells, interferon-stimulated B cells and germinal center-associated B cells, and described the phenotype, gene enrichment, cell–cell communication, biological processes. We utilized immunohistochemistry (IHC) and immunofluorescence (IF) to describe TLSs morphology in MIBC.

**Results:**

The interferon-stimulated B-cell subtype (B-ISG15) and germinal center-associated B-cell subtypes (B-LMO2, B-STMN1) were significantly enriched in MIBC. TLSs in MIBC exhibited a distinct follicular structure characterized by a central region of B cells resembling a germinal center surrounded by T cells. CellChat analysis showed that CXCL13 + T cells play a pivotal role in recruiting CXCR5 + B cells. Cell migration experiments demonstrated the chemoattraction of CXCL13 toward CXCR5 + B cells. Importantly, the infiltration of the interferon-stimulated B-cell subtype and the presence of TLSs correlated with a more favorable prognosis in MIBC.

**Conclusions:**

The study revealed the heterogeneity of B-cell subtypes in MIBC and suggests a pivotal role of TLSs in MIBC outcomes. Our study provides novel insights that contribute to the precision treatment of MIBC.

**Supplementary Information:**

The online version contains supplementary material available at 10.1186/s12967-024-04860-1.

## Highlights

- Single-cell RNA sequencing analysis identified 12 B-cell subtypes and characterized the transcriptomic heterogeneity of B cells in muscle-invasive bladder cancer (MIBC).
- The interferon-stimulated B-cell subtype (B-ISG15) and germinal center-associated B-cell subtypes (B-LMO2, B-STMN1) were significantly enriched in muscle-invasive bladder cancer.
- TLSs in MIBC are distinguished by the presence of a central region of CD19+ B cells forming a follicular structure characteristic of a germinal center and surrounded by T cells.
- Interferon-stimulated B-cell subtype and tertiary lymphoid structures are associated with favorable survival in MIBC patients.

**Supplementary Information:**

The online version contains supplementary material available at 10.1186/s12967-024-04860-1.

## Introduction

Bladder cancer ranks as the tenth most frequently diagnosed cancer globally [1]. Muscle-invasive bladder cancer (MIBC) involves invasion of the detrusor muscle layer or concurrent metastasis and is a highly aggressive disease with a poor prognosis [2–4]. The tumor microenvironment (TME) is a multifaceted and dynamic entity that exhibits high complexity in terms of cell types and interactions [5, 6]. Current studies indicate B cells are the primary effectors of humoral immunity in the TME and play a significant role in the regulation of the antitumor immune response [7–9]. CD19 + tumor-infiltrating B-cells function as APCs within the TME of MIBC, facilitating the activation of CD4 + tumor-infiltrating T-cells (TIT) [10]. IHC analysis of bladder cancer confirmed the presence of CD20 + B cells, but the role of these cells has not been addressed [11]. In melanoma, tumor-induced plasmablast-like-enriched B cell could maintain tumor inflammation and recruit CD8 + T cells [12], and tumor-associated CD20 + B cells are surrounded by CD4 + T cells, which is associated with improved survival [13]. Single-cell data analysis of colorectal cancer (CRC) patient samples revealed two main B-cell populations: CD20 + B cells and CD138 + plasma cells. Among them, CD20 + B cells comprise memory B cells, naïve B cells, germinal center (GC) B cells and plasma cells [14]. Naïve B cells and memory B cells can facilitate antigen presentation to T cells, activating them to target tumor cells. In non-small cell lung cancer (NSCLC), naïve B cells have been shown to inhibit tumor growth [15]. However, the B-cell subtypes involved in MIBC and their functions are still not fully characterized.

Tertiary lymphoid structures (TLSs) are organized aggregates of immune cells that form postnatally in nonlymphoid tissues [16, 17]. The presence of B cells within TLSs has been observed in various cancers [13, 18, 19]. Analysis of single-cell transcriptome data from NSCLC patients revealed that CD20 + CD22 + ADAM28 + BIR (ICI-responsive B cells) cells in TLSs predicted and contributed to the response to checkpoint immunotherapy [20]. Single-cell sequencing was applied to analyze the immune cells of metastatic melanoma and renal cell carcinoma cohorts and revealed a positive correlation between B cells and TLSs and patient response to immunotherapy [16]. The spatial transcriptomics analysis of renal cell carcinoma (RCC) revealed that B cells were enriched in TLS + tumors. Notably, IGHG1 + and IGHA1 + plasma cells near TLSs migrated along CXCL12 + fibroblasts in the tumor [21]. TLSs present heterogeneity among different cancer types and patients, and chemokines play important roles in cell interactions in TLSs [22]. A thorough understanding of the TLSs formation mechanism and the cell interactions that occur within TLSs could reveal potential therapeutic targets of MIBC.

In this study, we utilized scRNA-seq to comprehensively understand the heterogeneity and function of B cells in MIBC. To better understand the formation mechanisms of TLSs in MIBC, we utilized IHC and IF to characterize TLSs morphology in patients with MIBC. Additionally, we investigated the clinical prognostic relevance of B cells and TLSs in MIBC. Our findings offer more pertinent information on MIBC.

## Materials and methods

### Patients and samples

Study procedures were approved by the Medical Ethics Committee of Guangxi Medical University, and only patients who provided signed informed consent for sample collection and data analysis were included in this study. Samples were obtained from the First Affiliated Hospital of Guangxi Medical University and the Affiliated Tumor Hospital of Guangxi Medical University. Twelve patients, including ten males and two females, were enrolled in this study (Additional file 2: Table S1). Five patients presented with MIBC, and three patients presented with NMIBC. Nonmalignant bladder tissue samples were collected from nine patients, and samples from three patients were analyzed in our previous study [23]. None of the patients received radiation, chemotherapy, or intracystic treatment before surgery.

### Sample collection and single-cell processing

After resection in the operating room, adjacent nonmalignant bladder tissue was isolated at least 3 cm away from the tumor. Fresh tumor tissue, adjacent nonmalignant bladder tissue, and lymph node samples stored in cold HBSS containing 1% penicillin‒streptomycin were transported to the laboratory within 20 min. The tumor was divided into two pieces, one primarily derived from the tumor core and the other derived from the tumor margin. Full-thickness samples of the tumor and adjacent nonmalignant tissues were generated with a surface diameter of 1 cm. After trimming fat, the samples were rinsed twice in cold DPBS, minced into pieces of approximately 1 mm^3^ on ice, transferred into 15 ml tubes, and digested at 37 °C with collagenase I (1.5 mg/ml), collagenase II (1 mg/ml), and DNase I (0.2 mg/ml) with manual shaking for 40 min. The lymph node samples were digested with collagenase IV (1 mg/ml) and DNase I. Fresh peripheral blood was collected prior to surgery, and then peripheral blood mononuclear cells (PBMCs) were isolated using lymphocyte separation medium (Human) (Solarbio). The dissociated cells were filtered through a 100 μm cell strainer in DPBS and collected by centrifugation at 300 × g and 4 °C for 3 min; the supernatant was discarded. The pelleted cells were suspended in 5 ml of red blood cell lysis buffer, incubated on ice for 5 min to lyse red blood cells, filtered through a 40 μm cell strainer, and centrifuged at 300 × g and 4 °C for 3 min. After washing with cold DPBS twice, the cell pellets were resuspended in 500 μl of DPBS supplemented with 1% fetal bovine serum (FBS). The cells were stained with 0.4% trypan blue, and the concentration of live cells was determined using a hemocytometer. Samples with a cell viability rate of greater than 80% were subsequently used for single-cell library construction. The entire procedure took approximately 3 h to complete.

### *10* × *library preparation and sequencing*

The single-cell suspensions were adjusted to a concentration of 1500 cells/µl, and a total of 22,000 cells/sample were loaded onto a chromium controller utilizing Chromium Single-Cell 3′ Reagent Kits (v3 chemistry). Samples were sequenced using Illumina Nova S6000 instruments.

### Single-cell RNA sequencing (scRNA-seq) data processing

FASTQ data generated from sequencing output were aligned and quantified to a human reference genome (GRCh38) using Cell Ranger software (pipeline version 3.1.0). An overview website and a file containing a features table, barcode table, and feature-barcode matrix were generated. The website summarized the sample information, which included the estimated number of cells, mean reads per cell, median genes per cell, median unique molecular identifier (UMI) counts per cell, and sequencing saturation. The feature-barcode matrix was converted to a Seurat object using the R package Seurat (version 3.1.1) [24]. Low-quality cells were excluded based on the following criteria: mitochondrial gene percentage > 15%, < 300 genes/cell, and > 7500 genes/cell for tissue samples or > 5000 genes/cell for PBMC samples. Cells were retained based on the following criteria: mitochondrial gene percentage < 15% and 300 < genes/cell < 4000. After filtering, the feature-barcode matrix for each sample was normalized with the “NormalizeData” function with default parameters (“LogNormalize” method and scale.factor = 10,000), and the top 2,000 highly variable genes (HVFs) were identified using the “FindVariableFeatures” function (“vst” method). Then, the feature-barcode matrix was scaled by the “ScaleData” function, and the doublets for each sample were identified using the R package DoubletFinder (assuming a 5% doublet formation rate) [25].

### Sample aggregation, dimensionality reduction, and clustering

After removing doublet cells, the filtered feature-barcode matrix of all samples was merged. To remove batch effects across different samples, identification of the anchor correspondences of the merged data was performed with the “FindIntegrationAnchors” function, and the first 50 dimensions were used for calculation. Subsequently, these computed anchors were used for integration of the merged data with the “IntegrateData” function. The integrated data were normalized, and then the HVFs were identified in Seurat. Variables “percent.mito” and “nCount_RNA” were regressed out with the “ScaleData” function, and principal component analysis (PCA) was performed using the “RunPCA” function. The top 50 principal components were used for dimensionality reduction to visualize cells with the “RunTSNE” function, and the major clusters were identified with the “FindNeighbors” and “FindClusters” functions with a resolution of 1.4 to obtain a relatively good result. Notably, cells expressing dual-lineage genes were excluded from downstream analysis.

### Identification of cluster marker genes

Differential gene expression analysis was performed using the Seurat “FindAllMarkers” function: marker genes for major clusters or cell subtypes detected in at least 25% of cells were sorted by mean log2 (fold change) and filtered using a minimum log2 (fold change) value of 0.25. A gene was considered significantly different when the adjusted *p* value < 0.05 (adj.*p*). The top 100 differentially expressed genes in each major cluster or subtype were analyzed for biological process enrichment using Metascape [26].

### Single-cell regulatory network inference and clustering (SCENIC) analysis

To assess the regulatory characteristics of cell clusters, SCENIC was employed to analyze gene regulatory networks as previously described [27]. In brief, coexpression modules were inferred by the “runGENIE3” function, and potential direct binding targets (regulons) of transcription factors were identified using the human motif database of 10 kb around the transcription start site (TSS) for RcisTarget. Then, regulon activity in cell clusters was evaluated via AUCell and averaged. A heatmap for each regulon cluster was generated with the R package heatmap.

### RNA velocity analysis

The velocyto.R program was used for RNA velocity analysis [28]. First, spliced/unspliced reads for each sample were annotated with velocyto.py using the possorted_genome_bam.bam file, which was generated by Cell Ranger and then saved in a.loom file. Second, the.loom files for each sample were loaded into R and combined to generate count tables that contained spliced and unspliced reads. Next, the cells in the bottom 0.5% of the total unspliced transcript count were filtered out. Third, genes with an average spliced variant expression of lower than 0.2 or an average unspliced variant expression of lower than 0.05 in at least one cluster were removed. Finally, the arrows indicating RNA velocity information were embedded in the tSNE plot obtained from Seurat.

### Cell‒cell interaction analysis

Ligand‒receptor (L-R) interaction scores were calculated according to the R package CellChat [29]. L-R pairs with an interaction score between B cells and T-cell subtypes are presented.

### Immunofluorescence (IF), immunohistochemical (IHC), and hematoxylin and eosin (H&E) staining

In brief, sections were deparaffinized, rehydrated, and washed with PBS (P1010-2, Solarbio), and antigen retrieval was then performed via a high-pressure heat repair process with sodium citrate buffer (catalog number C1032; Solarbio). Endogenous peroxidase activity was blocked with 3% H2O2 for 30 min. After incubation with blocking buffer (PBS with 10% goat serum) at room temperature for 15 min, the sections were incubated with a primary antibody in a humidified chamber at 4 °C in the dark overnight, washed 3 times, and stained with a secondary antibody in a humidified chamber at 4 °C in the dark. For IHC staining, an HRP-conjugated secondary antibody was used, and the sections were reacted with diaminobenzidine (DAB) prior to counterstaining with hematoxylin. For IF, nuclear staining was performed using DAPI, and the sections were mounted with antifade mounting medium. For H&E staining, sections were dewaxed and hydrated, and nuclei and cytoplasm were directly stained with hematoxylin and eosin. Then, sections were sealed with neutral gum. Images were acquired using Leica Microsystems (DMI8), fully integrated stimulated Emission Depletion (STED) systems (Leica SP8), or a NanoZoomer S60 Digital slide scanner (C13210, Hamamatsu, Japan).

### Construction and validation of a prognostic value of B-cell subtypes

Intersected The Cancer Genome Atlas-Bladder Urothelial Carcinoma (TCGA-BLCA) with the characteristic gene sets of each B-cell subtype to obtain the intersection genes, univariate Cox analysis was performed using the TCGA-BLCA database aiming to identify genes that exhibit significant associations with overall survival (OS). Next, genes were screened for model construction using the Least Absolute Shrinkage and Selection Operator (LASSO) analyses. According to cut-of, patients were divided into high-level and low-level groups. Survival curves were visualized by the R packages “survival”. The validity of the prognostic model was confirmed using the IMvigor210 dataset [30].

### Survival analysis of TLS

The “survival analysis” module of Gene Expression Profile Interaction Analysis version 2 (GEPIA2) (http://gepia2cancer-pku.cn/) was used to construct Kaplan‒Meier plots of MS4A1 (CD20), CD3D, LMO2, and AICDA in TCGA-BLCA cohort. Furthermore, the findings were validated using the IMvigor210 dataset, and survival curves were generated using the R package “survival.”

### Chemotaxis assays

Chemotaxis assays were performed using 24-well transwell plates with 5 µm pores (Corning), following the guidelines provided by the manufacturer. Recombinant human CXCL13 (MCE, 1 μg/ml) was added to the bottom wells, B cells (1 × 10^5^) or B cells (1 × 10^5^) were pretreated with the CXCR5 antagonist ML339 (MCE, 40 μg/ml) for 30 min and placed in the upper wells. The cells were allowed to migrate for 12 h at 37 °C in 5% CO2. Migrating cells from the lower chamber were collected and counted.

## Results

### Extensive infiltration of B cells in MIBC

**Fig. 1:**
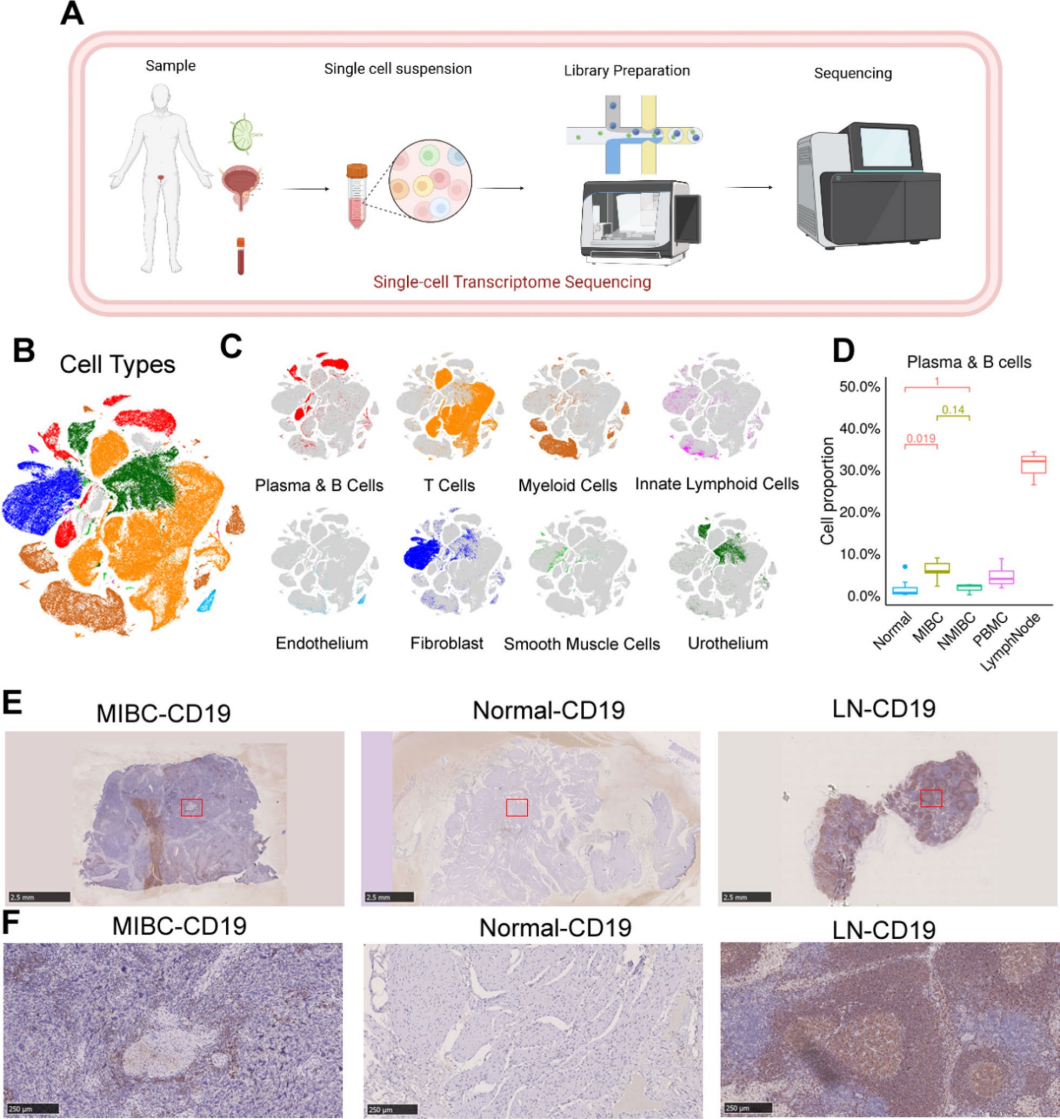
Single-cell transcriptional profiles of bladder cancer tissues and adjacent lymph nodes. **A** Schematic of the workflow. **B** The t-distributed stochastic neighbor embedding (tSNE) plot displays the cellular profiles of individual cells, which have been characterized by their dominant cell types. **C** tSNE plot of single cells profiled by dominant cell types (B cells). **D** Box plot showing the proportion of B cells and plasma cells from MIBC, NMIBC, normal tissue, PBMC, and lymph node samples. MIBC, n = 7; NMIBC, n = 3; Normal, n = 9; PBMC, n = 4; lymph nodes, n = 3. **E**, **F** Immunohistochemical staining of CD19 showing B-cell infiltration in tumor (left), normal tissue, and lymph node (right) samples. Scale bar: 2.5 mm in panoramic images and 250 μm in magnified images

As shown in the flowchart (Fig. 1A), to delineate the comprehensive transcriptional atlas of bladder cancer, tumor samples, adjacent normal tissue, lymph nodes and peripheral blood samples were surgically obtained from 12 bladder cancer patients (MIBC, n = 7; NMIBC, n = 3; normal, n = 9; PBMC, n = 4; lymph nodes, n = 3) (Additional file 2: Table S1). The specimens underwent 3'-end single-cell RNA-seq processing utilizing the 10 × Genomics platform. Then, preliminary quality control was performed. A total of 179,769 cells, including 55,721 single cells from the MIBC samples, 19,887 single cells from the NMIBC samples, 51,783 single cells from the adjacent nonmalignant tissue samples, 33,816 peripheral blood mononuclear cells (PBMCs), and 18,562 single cells from the lymph node samples were obtained. We successfully annotated cell types, including epithelial cells, fibroblasts, smooth muscle cells, endothelial cells, T cells, myeloid cells and B cells (Fig. 1B, C).

We observed massive infiltration of B cells in MIBC (Fig. 1D). To verify this at the protein level, we performed IHC staining for the B-cell marker CD19 to compare B-cell infiltration in MIBC tissues and surrounding normal bladder tissues in clinical specimens. The infiltration level of CD19 + B cells was significantly higher in MIBC tissues (Fig. 1E). A distinct honeycomb-like area of B-cell infiltration was found in MIBC. This region was not observed in the surrounding normal bladder tissue (Fig. 1F). The high level of B cell infiltration suggests that B cells play an important role in the TME of MIBC.

### Heterogeneity of B cells in MIBC

**Fig. 2:**
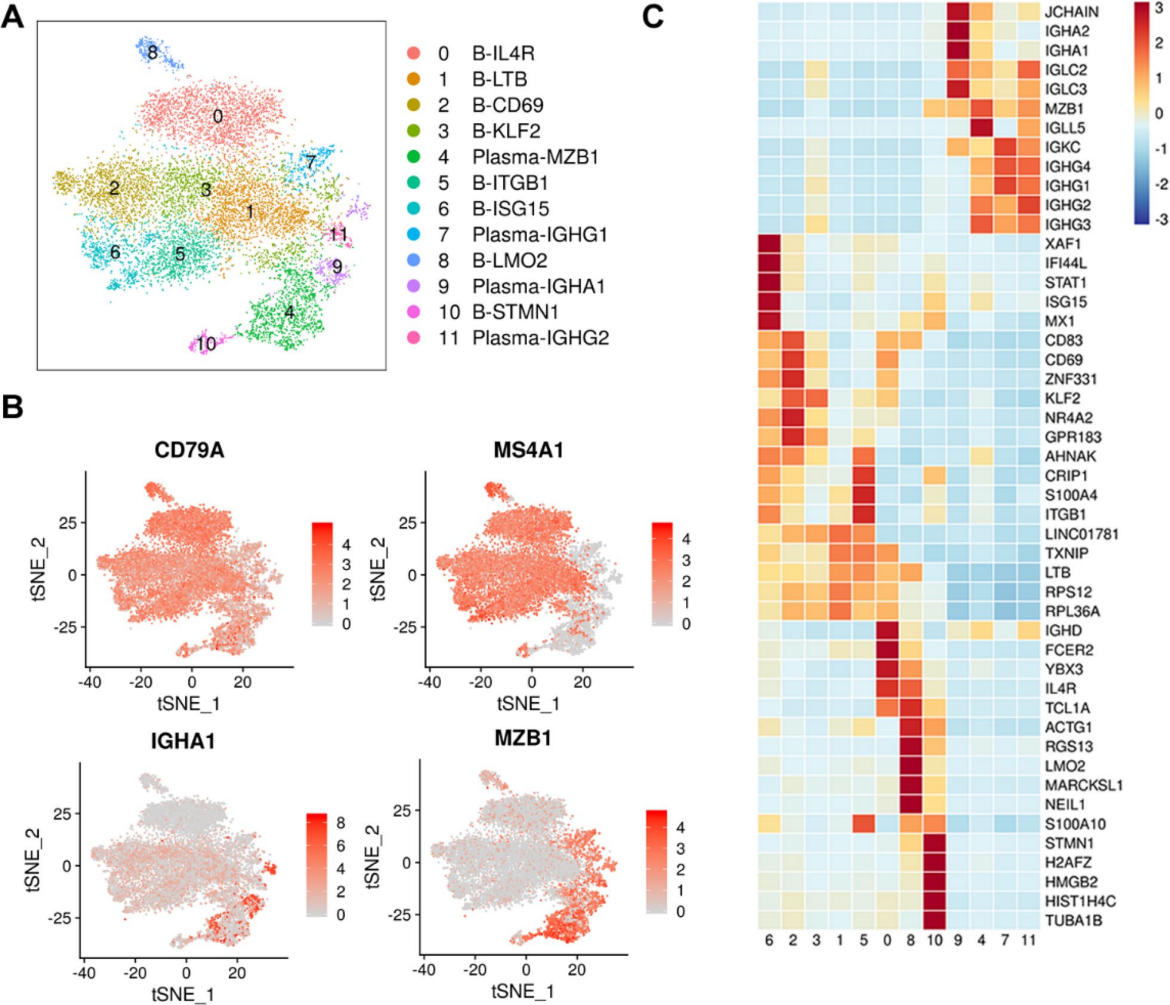
B cells in MIBC. **A** tSNE plot showing the subclustering of 11,612 B cells from 12 patients with bladder cancer. Each cluster is shown in a different color. Twelve clusters are shown in each plot. **B** The tSNE map shows the distribution of B-cell characteristic genes *CD79A* and *MS4A1* and plasma cell characteristic genes *IGHA1* and *MZB1*. **C** Heatmap showing the expression levels of functional markers of B-cell subtypes

To characterize the transcriptomic heterogeneity of B cells in MIBC, a total of 11,612 cells were obtained by reclustering B-cell groups separately extracted from the total original data. After extracting all B cells (from the marker genes *CD79A*, *MS4A1*, *IGHA1*, and *MZB1*) for further subclustering analysis, we detected 4 plasma cell subtypes and 8 B-cell subtypes (Fig. 2A, B).

To further describe the diversity of B cells in MIBC, we refer to B-cell-specific gene markers reported in previous articles. Plasma cell populations showed enrichment of several immunoglobulin transcripts (*IGHG1*, *MZB1*, and *IGHA1*). B-LTB subtypes, B-KLF2 subtypes and B-ITGB1 subtypes expressed higher levels of apoptosis-related genes (*KLF2*, *TXNIP*, *RPS12*) and were recognized as apoptotic B cells. The B-STMN1 subtype highly expressed the proliferation-related gene *STMN1*, suggesting that there is a subtype of proliferating B cells in the TME of MIBC that affects tumor immunity. B-CD69 subtypes highly express *CD69*, and a single-cell RNA sequencing study of melanoma tissues revealed that CD69 + B cells were associated with the tumor response to immune checkpoint inhibitors. Of interest, B-IL4R subtypes highly expressed mature B-cell characteristic genes (*IGHD*) and highly expressed *IL4R*, which has been reported to be highly expressed in naïve B cells, suggesting that B-IL4R may be in the intermediate state of B-cell development and transformation (Fig. 2C). The heterogeneity of B cells in the TME of MIBC prompted us to further explore the role of B cells in MIBC.

### Enrichment of the interferon-stimulated B-cell subtype in MIBC

**Fig. 3:**
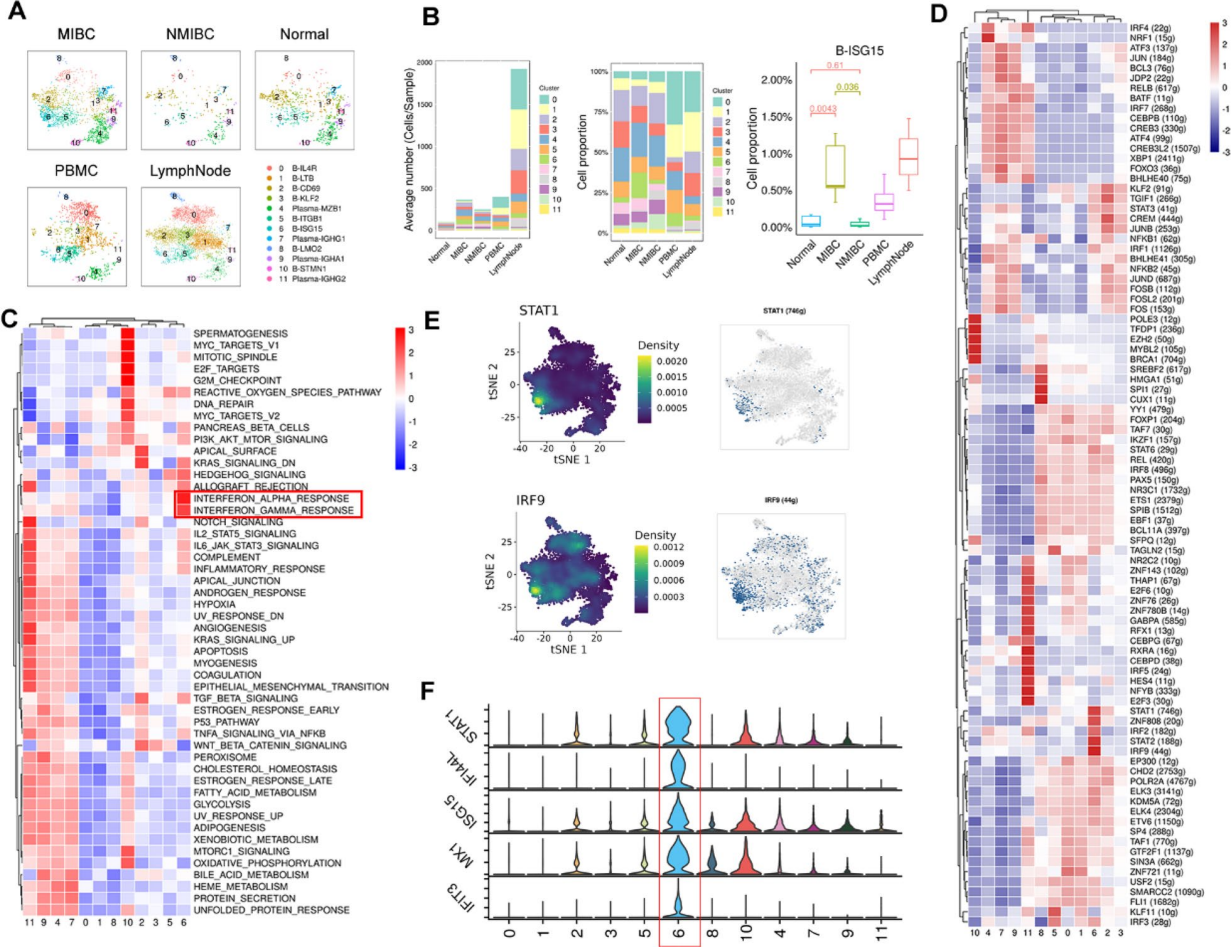
Interferon-stimulated B cells are enriched in MIBC. **A** tSNE plots of B cells in MIBC; n = 7; NMIBC, n = 3; normal, n = 9; PBMC, n = 4; lymph nodes, n = 3. Each cluster is shown in a different color. **B** The bar plots depict the mean cell count of each B-cell subtype in each sample on the left. In the middle, the bar plots illustrate the proportion of each B-cell subtype in each sample. On the right, a box plot displays the proportion of B-ISG15 in each sample.; MIBC, n = 7; NMIBC, n = 3; Normal, n = 9; PBMC, n = 4; lymph nodes, n = 3. **C** Heatmap shows the GSEA score of a set of hallmark genes enriched in B-cell subtypes. **D** Heatmap showing the activity of SCENIC transcription factor regulons in each B-cell subtype. **E** Expression levels of *STAT1* and *IRF9* marker genes illustrated in tSNE plots in each B-cell subtype. **F** The violin plots depict the expression patterns of IFN-γ genes, colors as in (A)

To further explore the heterogeneity of B-cell subtypes, we performed a cluster analysis of B cells derived from different tissues for each cluster (Fig. 3A). In addition, we counted the number and percentage of each B-cell subtype from different tissues. Although all 12 B-cell subtypes were present in both MIBC and NMIBC, the level of infiltration for each of these B-cell types was different, reflecting differences in the B cell infiltration profiles for each stage of bladder cancer progression. Notably, the proportion of B-ISG15 subtype cells in MIBC was significantly higher than that in NMIBC (Fig. 3B).

The ISG15 subtype highly expressed the interferon-stimulated gene *STAT1* and interferon-stimulating gene *ISG15* (Fig. 2C). Based on hallmark gene set enrichment analysis, we identified B-ISG15 as an interferon-stimulated B-cell subtype. B-ISG15 was enriched in interferon-α and interferon-γ response gene sets. We also found that B-ISG15 was enriched in the IL2 STAT5 signaling and IL6 JAK STAT3 signaling gene sets, and IL-6/JAK/STAT3 signaling drives tumor cell proliferation, survival, invasion, and metastasis while inhibiting the antitumor immune response (Fig. 3C).

B-ISG15 exhibited high enrichment of transcription factors in the interferon pathway (Fig. 3D) and of STAT1- and IRF9-regulated genes (Fig. 3E) and high expression levels of downstream genes involved in interferon signaling, including *STAT1*, *IFI44L*, *ISG15*, and *MX1* (Fig. 3F). In general, B-ISG15 was significantly enriched in MIBC, confirming that strong interferon signaling is a prominent feature of the TME in MIBC.

### Presence of germinal center-associated B cells in MIBC

**Fig. 4:**
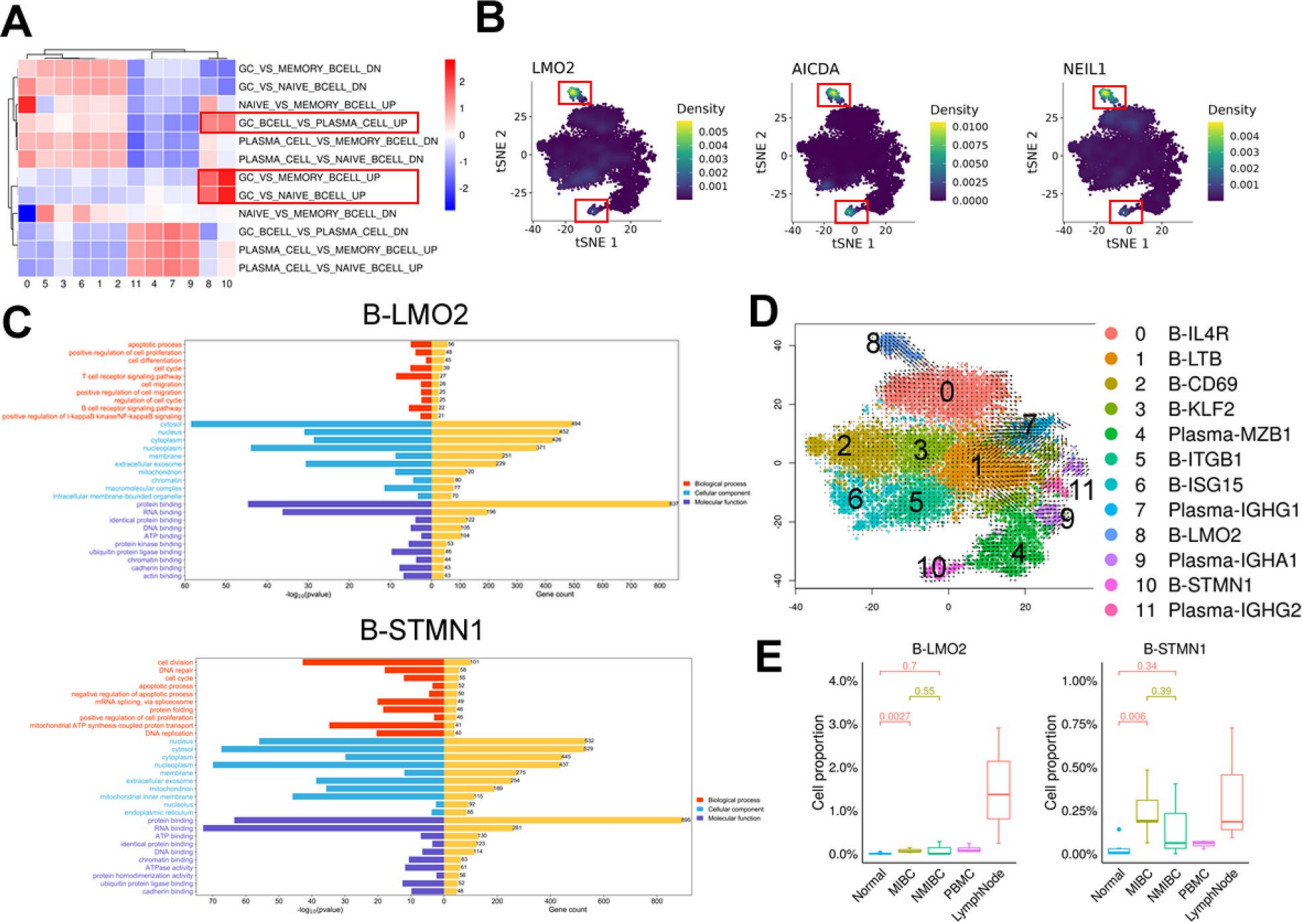
Germinal center-associated B-cell subtypes present in MIBC. **A** Heatmap shows GSVA scores of enriched germinal center-associated gene sets. **B** tSNE plot showing the expression densities of the *LMO2, AICDA*, and *NEIL1* genes in B-cell subtypes. **C** The Gene Ontology (GO) terms of genes that exhibited significant enrichment in B-LMO2 (top) and B-STMN1 (bottom) were determined through the application of Fisher's test for statistical analysis. **D** RNA velocity of B-cell subtypes. It shows the transformation of B-cell subtypes. **E** Box plot showing the proportions of B-LMO2 (left) and B-STMN1 (right) in MIBC, NMIBC, normal tissue, PBMC, and lymph node samples

We performed GSVA analysis of enriched germinal center-related gene sets and gene enrichment analysis in B-cell subtypes, and two subtypes of GC B cells (B-LMO2, B-STMN1) were identified (Figs. 2C, 4A). B-LMO2 showed high enrichment of germinal center genes *LMO2* [31], *AICDA* (activation-induced cytosine deaminase) [32] and *NEIL1* (which facilitates the repair of oxidative DNA damage) [33], and proliferating B cells B-STMN1 also showed high enrichment of germinal center genes (Fig. 4B).

To further characterize the functions of genes enriched in the germinal center-associated B-cell subtypes, we performed Gene Ontology (GO) enrichment analysis. We noticed that B-LMO2 was related to “positive regulation of NF-κB signaling” and “T-cell receptor and B cell receptor signaling pathway”, and apoptosis is one of the characteristics of GC cells [34]. “Positive regulation of cell proliferation” “cell differentiation” and “positive regulation of cell migration” were also enriched in B-LMO2 cells, suggesting potential associations with other cellular interactions. B-STMN1 was related to “cell division”, “positive regulation of cell proliferation” and “cell division”, further confirming that this subtype is related to B-cell proliferation (Fig. 4C).

To infer the differentiation trajectories of B cells in MIBC, we performed RNA velocity analysis. This analysis predicted that plasma-MZB1, plasma-IGHG1, plasma-IGHA1 and plasma-IGHG2 likely originate from proliferative B cells (B-STMN1) and that B-IL4R subtype cells could develop into B-LMO2 subtype cells (Fig. 4D), implying that naïve B-cell differentiation is induced to promote the formation of germinal center cells and that germinal center proliferative B cells differentiate into other types of B cells and play a role in the TME. B-STMN1 was in a proliferative state and recognized as a centroblast found in the dark zone, and B-LMO2 showed high enrichment of germinal center genes constituting centrocytes in the light zone [35]. Interestingly, B-STMN1 and B-LMO2 were enriched not only in lymph nodes but also in MIBC, implying the possible presence of TLSs (Fig. 4E).

### Identification of TLSs in MIBC

**Fig. 5:**
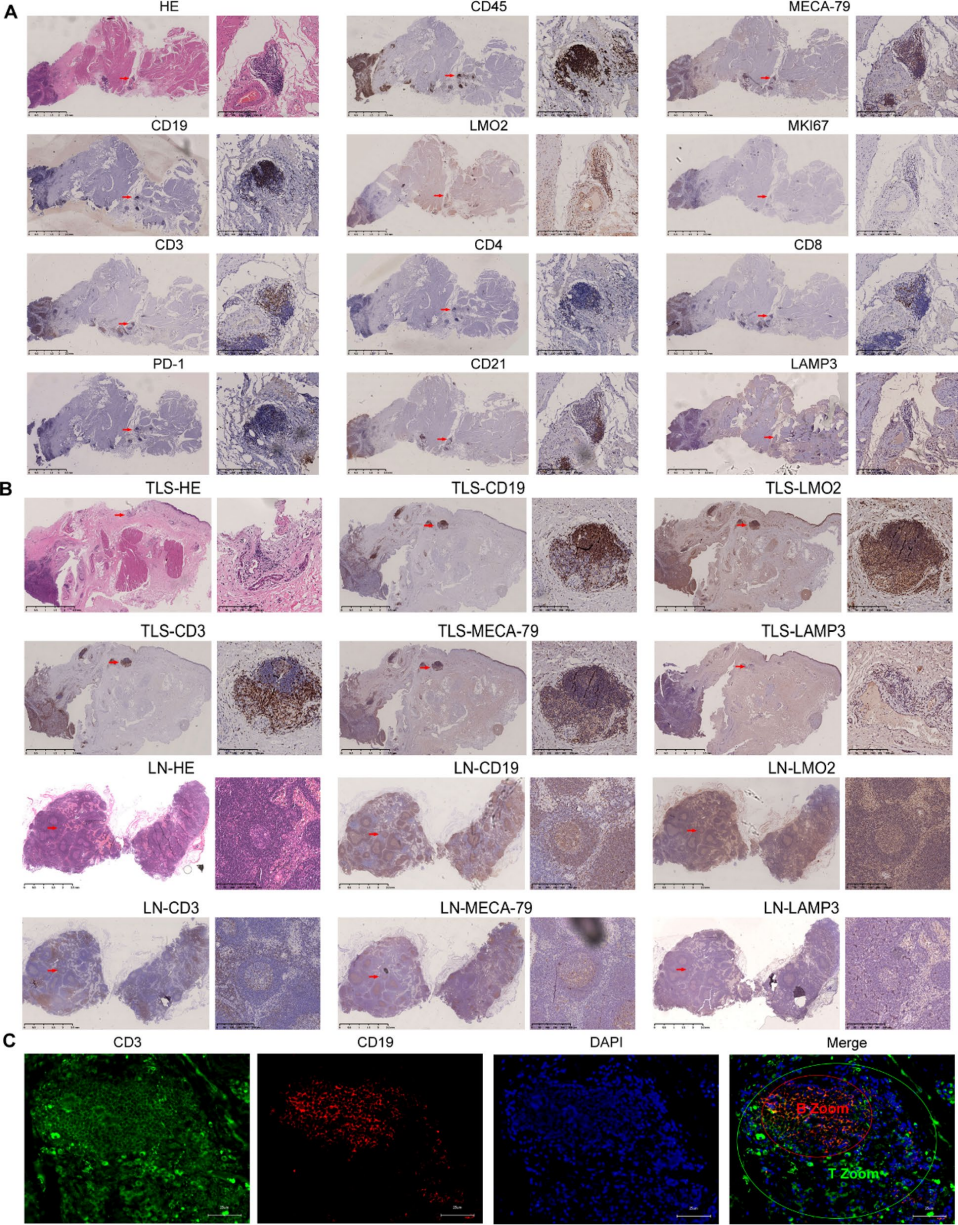
TLSs in MIBC. **A** HE staining shows TLSs (red squares) in MIBC, and IHC staining shows the following markers of TLSs (red squares): CD45, CD19, CD3, MKI67, CD4, CD8, PD-1, CD21, LAMP3, MECA79A and LMO2. Scale bar: 2.5 mm in panoramic images and 100 μm in magnified images. **B** H&E staining and IHC for the indicated markers for representative LNs (two columns below) and TLSs (two columns above). Scale bar: 2.5 mm in panoramic images and 100 μm in magnified images. **C** mIF for the indicated markers for representative TLSs. The following markers are shown in individual and merged channels: CD3 (green), T-cell marker; CD19 (red), B-cell marker; DAPI (blue), nuclear marker. Scale bar: 25 μm

To further verify the presence of TLSs in MIBC, HE staining was performed and revealed that TLS-like structures were present in tumor tissues. IHC staining in tumor tissues confirmed that there was cell aggregation near blood vessels in the stroma of the MIBC tumor margin, and CD45 staining confirmed immune cell aggregation. We identified a follicular structure characteristic of a germinal center (CD19, LMO2, MKI67). CD19 staining showed that B cells formed internal clumps, and a small number of LMO2 + B cells were present. MKI67 staining showed that proliferation genes were positive in B-cell clusters. At the same site, staining of the T-cell marker CD3 showed that T cells were enriched in the periphery of B cells, forming a typical T-cell area, and different types of CD4 + and CD8 + T cells could be seen. PD-1 staining was positive, suggesting that some Tregs were present (CD3, CD8, CD4, PD-1); CD21 + DCs showed a mosaic distribution pattern in the T-cell area, and some LAMP3 + DCs were present. MECA79, a marker of high endothelial venules (HEVs), was also found to be expressed around TLSs (Fig. 5A).

To clarify the similarities and differences between the TLSs in MIBC and the germinal center region in lymph nodes, we compared and analyzed the two. B cells are surrounded by T cells to form distinct T-cell and B-cell zones (CD19, CD3). B cells form a follicular structure characteristic of a germinal center (CD19, LMO2). LAMP3 + DCS were interspersed among T cells, and MECA79 + HEVs were present near GC. We clearly observed blood vessels around the TLSs in MIBC. The TLSs in MIBC was highly similar to the germinal center zone in lymph nodes (Fig. 5B). The presence of TLSs was further confirmed by IF staining. Indeed, a germinal center-like structure surrounded by B cells (CD19 + zone) and T cells (CD3 + zone) was evident, indicating an interaction between T cells and B cells (Fig. 5C).

### Recruitment of CXCR5 + B cells to form a B-Cell Zone by T cells expressing CXCL13 in MIBC

**Fig. 6:**
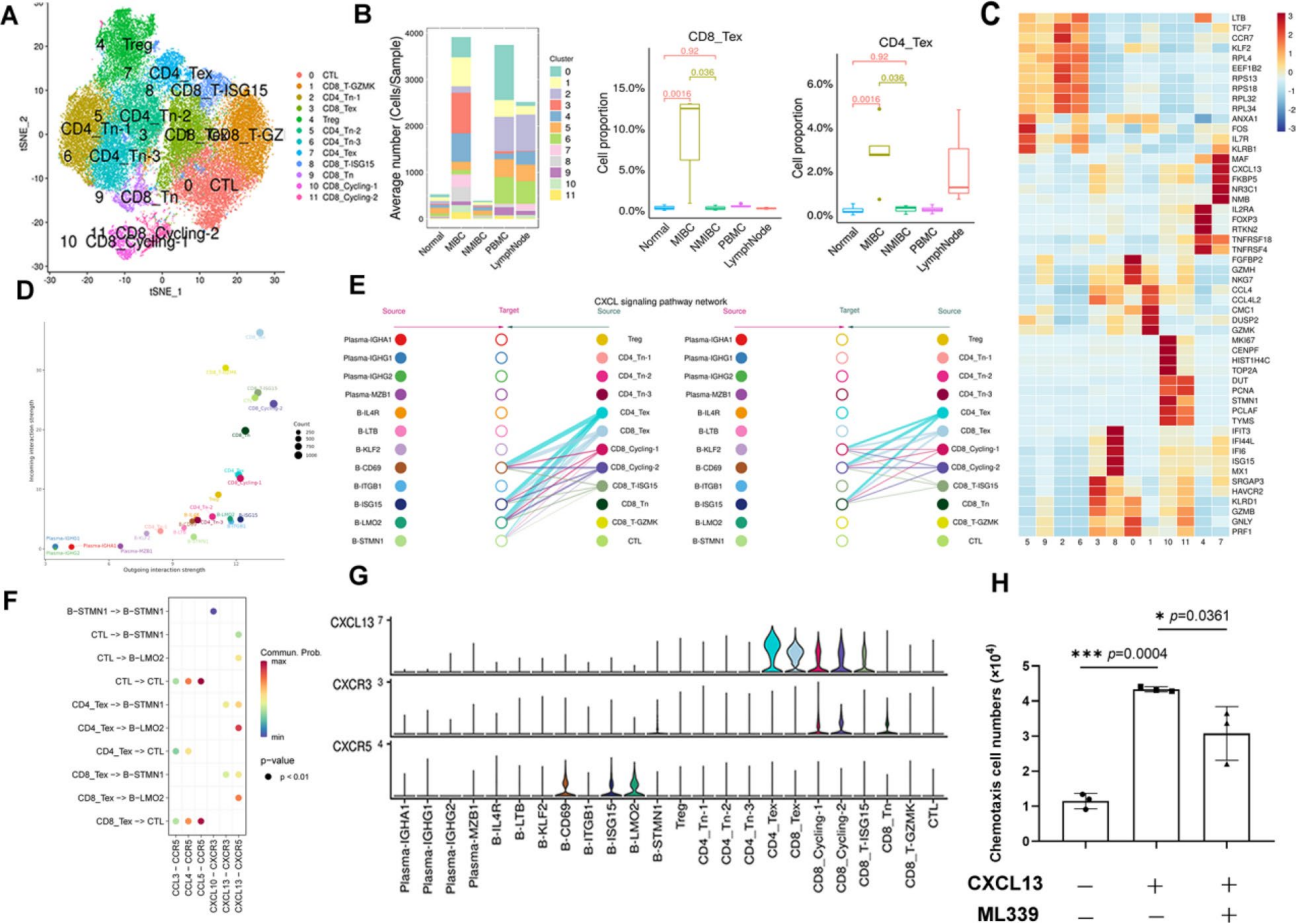
Transcriptome analysis revealed that T cells recruit B cells via CXCL13-CXCR5 in MIBC. **A** tSNE plot showing T-cell subtypes. **B** Average cell numbers of T-cell subtypes in original samples (left). Box plot showing the proportions of CD8_Tex (middle) and CD4_Tex (right) cells in the original samples. **C** Heatmap indicating the scaled expression of T-cell marker genes. **D** Bubble plot indicating cell roles: The color of the dots represents different cell groups, the size of the dots is proportional to the number of ligands and receptors inferred for each cell group, and the x and y axes indicate the strength of the cell group as a signal sender and receiver, respectively. **E** Hierarchy diagram: “Source” represents the cell class that sends the signal, “target” represents the cell class that receives the signal, and the circle color represents the cell class. **F** The signaling pathway relationship to the bubble diagram shows significant interactions between the ligand‒receptor (L-R) pairs of subpopulations. **G** The violin diagram shows the expression levels of genes involved in signaling pathways. **H** Migration of CXCR5 + B cells treated with (middle) or without (left) CXCL13 and the CXCR5 antagonist (right)

To further understand the interaction between T cells and B cells in TLSs, we clustered the T-cell groups extracted from the original data. Twelve T-cell subtypes were identified (Fig. 6A), and each subtype was annotated based on marker gene expression (Fig. 6C). T-cell infiltration in MIBC was higher than that in normal and NMIBC tissues. Notably, exhausted T cells (Texs) accumulated in MIBC (MIBC 49.02%, NMIBC 20.06%, and normal 11.33%), while Tregs were observed primarily in NMIBC (MIBC 12.82%, NMIBC 28.52%, and normal 11.44%), indicating a difference in the immune microenvironment between MIBC and NMIBC (Fig. 6B).

CellChat was used to compare the intensity of efferent and afferent interactions between T cells and B cells in tumors and to analyze the cell subtypes with significant difference in sent or received signals. The intensity of the signals emitted and received by most T-cell subtypes was very strong (CD8_Tex, CD8_T-GZMK, CD8_T-ISG15, CTL). B-LMO2, B-ISG15 and B-IL4R emitted and received strong signals (Fig. 6D). T cells play a role in the B-cell signal transduction pathway mainly through CXCL. According to network centrality analysis, CD8_Tex and CD4_Tex are the most important sources of CXCL ligands for B-CD69, B-ISG15, and B-LMO2 (Fig. 6E, Additional file 1: Fig S1A).

We further analyzed cell‒cell interactions in the tumor mass by calculating interaction scores for a set of ligand‒receptor (L-R) pairs. CD8_Tex and CD4_Tex were predicted to interact with B-LMO2 via CXCL13-CXCR5 interactions, and CD8_Tex and CD4_Tex interacted with B-STMN1 via CXCL13-CXCR5 and CXCL16-CXCR3 interactions (Fig. 6F, Additional file 1: Fig S1B). We observed that CD8_Tex and CD4_Tex highly expressed CXCL13, and CXCR5 was highly expressed in B-CD69, B-ISG15, and B-LMO2 cells (Fig. 6G, Additional file 1: Fig S1C). Based on these results, we suggest that in MIBC, B cells expressing CXCR5 are recruited by T cells expressing CXCL13. Clearly, CXCL13 induced chemotaxis in CXCR5 + B cells, while the CXCR5 antagonist blocked this effect (Fig. 6H).

### Prognostic value of B-cell subtypes and tertiary lymphoid structures in MIBC

**Fig. 7:**
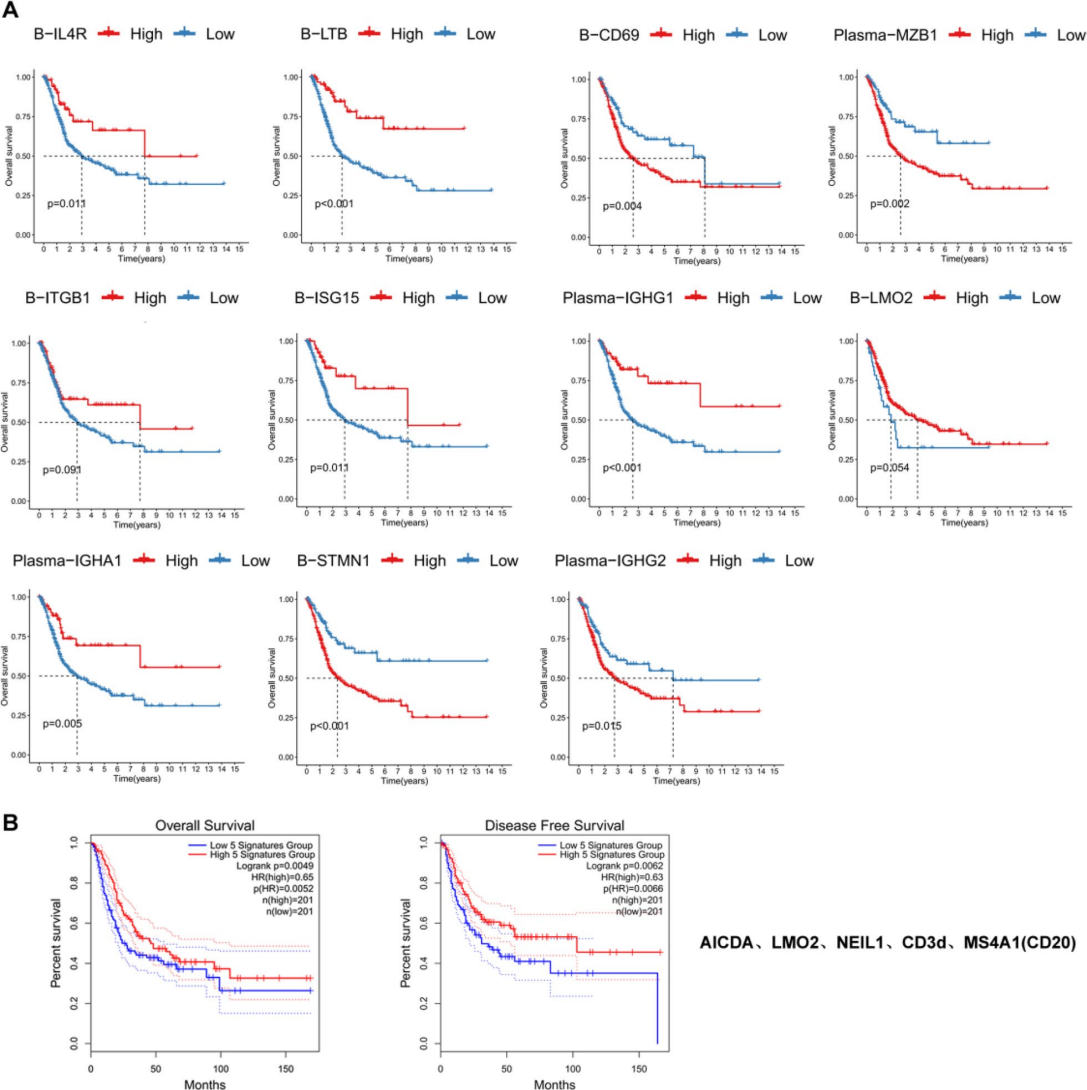
Prognostic value of B-cell subtypes and TLSs in MIBC. **A** The Kaplan‒Meier method was used to analyze the effect of different B-cell subtypes on the survival rate of patients with MIBC (*p* < 0.05). **B** The Kaplan‒Meier method was used to analyze the effect of *MS4A1 (CD20), CD3d, LMO2, AICDA* and *NEIL1* gene combination expression on the survival rate of patients with MIBC (*p* < 0.05)

To determine the impact of individual B-cell subtypes on the prognosis of patients with MIBC, we developed a risk prognostic model. The genes identified through LASSO analysis within B-cell subtypes are provided in Additional file 3: Table S2. The infiltration of B-STMN1, B-CD69, Plasma-MZB1 and Plasma-IGHG2 subtypes was found to be inversely correlated with the OS of patients diagnosed with MIBC, the presence of these B-cell subtypes was significantly associated with a poorer prognosis in patients with MIBC (*p* < 0.05). Notably, a positive correlation was observed between the infiltration levels of the plasma-IGHG1, plasma-IGHA1, B-ISG15, B-LTB and B-IL4R subtypes and the overall survival rates of patients diagnosed with MIBC, these B-cell subtypes exhibited favorable prognostic significance in patients with MIBC (*p* < 0.05). There was no observed correlation between the infiltration of B-LMO2 and B-ITGB1 subtypes and the OS of patients with MIBC (Fig. 7A). We performed validation on the IMvigor210 dataset, B-ISG15, B-IL4R, plasma-IGHA1 and plasma-IGHG1 subtypes exhibited favorable prognostic significance in patients with MIBC, and the B-LMO2 and B-ITGB1 subtypes exhibited a consistent trend across both databases (Additional file 1: Fig S2A).

The top 50 differentially expressed genes of germinal center-related B-cell subtypes were put into the TCGA database for comparison, and the expression of *LMO2*, *AICDA* and *NEIL1* in bladder cancer was observed (Additional file 1: Fig S3A). The expression levels of *MS4A1*, *LMO2* and *AICDA* did not have a significant impact on the OS rate or disease-free survival (DFS) rate of TCGA BLCA patients (*p* < 0.05). *NEIL1* expression levels showed a positive correlation with both OS and DFS, while *CD3d* expression levels exhibited a positive correlation solely with DFS (*p* < 0.05) (Additional file 1: Fig S3B). Then, we combined these five genes for survival analysis and found that patients survived longer when the five genes were highly expressed at the same time (*p* < 0.05) (Fig. 7B), suggesting that TLSs exhibit a protective effect against MIBC. The prognostic value of TLS was confirmed using the IMvigor210 dataset (Additional file 1: Fig S2B).

## Discussion

Tumor-infiltrating lymphocytes (TILs) are widely acknowledged as crucial factors in the regulation of tumor progression. Currently, extensive research has investigated TIT cells, and the significance of B cells in cancer has gained increasing interest. Herein, we utilized single-cell RNA sequencing to characterize the B-cell landscape of bladder cancer patients with high resolution. Our analysis identified 8 B-cell subtypes and 4 plasma cell subtypes. We observed massive infiltration of B cells and plasma cells in MIBC. The infiltration of plasma cells into the tumor suggests the activation of the immune system and the antitumor state in NSCLC [36]. High levels of B cells and/or plasma cell infiltration into the tumor are associated with increased aggressiveness of bladder cancer [37]. However, the infiltration of B cells in the TME is associated with a good prognosis in the majority of tumors [38, 39]. Regulatory B cells (Breg) have been reported to play a role in cancer immunity [39], yet Breg cells were not detected in our single-cell sequencing data. Naïve B cells have been identified in human tumors [15, 40], and have shown an association with improved overall survival and disease-free in triple-negative breast cancer [41]. In our study, we identified B-IL4R as exhibiting characteristics similar to those of naïve B cells and observed an association with prolonged OS in patients with MIBC. Previous studies have demonstrated an enrichment of proliferative B cells in both TLSs and tumors responsive to immunotherapy, highlighting the potential relevance of TLS formation and activity in certain tumor [13, 16]. In our investigation, we observed the presence of proliferative B cells (B-STMN1), characterized by a marked enrichment of GC genes. These results lend credence to the concept of TLS formation in MIBC. IHC staining showed that there was a higher level of infiltration of CD19 + B cells in MIBC than in NMIBC, indicating a large influx of B cells into the cancer nest and activation of the immune system at the tumor site [42]. In patients with MIBC enriched with CD19 + B cells, these cells can act as antigen presenting cells to activate CD4 + TIT cells in the tumor environment of MIBC [10].

Robust interferon signaling is a prominent feature of the TME in MIBC, and a high concentration of tumor interferon-gamma (IFN-γ) creates an immunosuppressive TME [43]. As intense T-cell exhaustion (Tex) infiltration occurs in MIBC, the CD8_Tex and CD4_Tex subtypes express high levels of IFN-γ in MIBC to regulate the activity of other immune subtypes. IFN-γ can upregulate PD-L1 in tumor cells and induce T-cell exhaustion [44]. In breast cancer, high IFN-γ expression and/or lack of IFNγR1 induced strong tumor rejection effects through IFN-γ-dependent or IFN-γ-independent signaling; however, moderate expression of IFN-γ may promote tumor escape and recurrence [45]. Additionally, abundant levels of interferon-stimulated T cells (CD8_T-ISG15) and B cells (B-ISG15) were observed in MIBC. The induction of BCL-6 through cell intrinsic mechanisms is promoted by B-cell IFN-γ receptor signaling, thereby contributing to the development of autoimmune germinal centers [46], indicating that IFN-γ signaling facilitated by Tex cell subtypes is correlated with TLSs generation in MIBC. The interferon-stimulated B-cell subtype was associated with longer OS in patients with MIBC, suggesting that IFN-γ may be a good prognostic marker. IFN-γ plays an important role in the regulation of the antitumor immune response and immunotherapy response [47–49]. In multiple tumor models, IFN-γ signaling pathways are inactive, but more sensitive to ICB [50]. However, its role as a predictor of clinical response to immunotherapy in MIBC needs to be further explored.

In the last few years, tumor-infiltrating B cells have been identified, and they are associated with a favorable prognosis in some cases [39, 51]. This study revealed two B-cell subtypes associated with germinal centers, and both cell types highly expressed *LMO2* [31] and *AICDA* [32], which has been reported to be a marker gene associated with the germinal center. The co-infiltration of B cells and CD8 + T cells is observed in MIBC, wherein characterized by a high proportion of PD-1 + TCF1 + progenitor T cells, naïve T cells and activated B cells [52]. We utilized immunofluorescence and IHC techniques to gain deeper insights into the structural characteristics of TLS in MIBC. TLSs are distinguished by the presence of a central region consisting of CD20 + B cells, encircled by CD4 + T cells and CD8 + T cells, resembling the lymphoid follicles observed in secondary lymphoid organs (SLOs) [17, 53, 54]. Different DC populations are selectively present in T-cell-rich areas near B-cell follicles; such DCs include CD21 + follicular dendritic cells (FDCs), which are of mesenchymal origin and play a key role in the selection of memory B cells during the GC response in SLOs [55]. MKI67 + proliferating B cells and follicular helper T cells were observed. HEVs positive for peripheral lymph node address protein (PNAd) provide a specialized vasculature associated with TLSs and serve as a pathway for lymphocyte recruitment [55]. H&E staining showed that TLSs were generated around blood vessels, constantly recruiting immune cells. TLSs were found in the invasive margin or the stroma but not in the tumor core. Multiple studies have shown that TLSs form a temporary military station, constantly recruiting T cells and B cells. Then, lymphocytes are transported to the tumor site to exert an immune response [16, 17, 19, 20, 22, 56–59], and an internal cancer-immunity cycle may be formed in these tissues.

However, the initiation and process of its formation is still unclear. We found by transcriptome analysis that CXCR5 + B cells (B-CD69, B-ISG15, B-LMO2) are recruited by T cells (CD8_Tex, CD4_Tex) expressing CXCL13 to form B-cell zones. Subsequently, cell migration experiments demonstrated the chemotactic attraction of CXCL13 toward CXCR5 + B cells. CXCL13, referred to as B-cell-attracting chemokine 1 (BCA-1), was observed to be secreted by follicular dendritic cells and CD4 + helper T cells (Th). The binding of CXCL13 to the CXCR5 receptor is crucial in orchestrating cellular migration across distinct regions of secondary lymphoid organs [60, 61]. A novel subpopulation of tumor-derived PD-1 + CXCR5^_^ CD4 + Th-CXCL13 cells has been discovered through research and plays a significant role in driving B cells into TLSs in nasopharyngeal carcinoma [62]. Our findings, supported by single-cell sequencing data as well as results from IHC and IF, allowed us to propose a mechanistic diagram (Additional file 1: Fig S4) illustrating this process.

The presence of TLSs in most tumors is associated with better prognosis and clinical outcome [2, 13, 17, 20, 63]. We evaluated the prognostic significance of TLS-related markers [*MS4A1 (CD20), CD3d, LMO2, AICDA* and *NEIL1*] in TCGA BLCA patients. TLSs exhibit a protective effect against bladder cancer and are associated with survival and prognosis. Previous studies have reported the discovery of TLSs in bladder cancer patient tissues and confirmed that well-developed TLSs are more common in aggressive high-grade MIBC tumors than in low-grade NMIBC [11]. The presence of TLSs as well as their components, such as follicular B cells, LAMP + mature DCs and HEVs, has been shown to correlate with better survival in many different tumor types [13, 16, 64–67]. In addition, multiple gene expression signatures associated with TLSs, including those of tumor-infiltrating T and B cells and mature TLSs. have shown prognostic value [68]. Therefore, TLSs may represent a promising therapeutic target for the treatment of MIBC.

These results suggest that verifying the biological functions of certain B-cell subtypes will help to further clarify their roles in MIBC. Since scRNA-seq only provides transcriptome data, the intracellular spatial information and cell‒cell interactions of TLSs in MIBC need to be further analyzed by spatial transcriptome analysis or multiplex immunofluorescence staining. Furthermore, T-cell secretion of CXCL13 has not been experimentally verified. The formation mechanism and potential role of TLSs still need to be addressed in future work.

In summary, single-cell transcriptome profiling of the heterogeneity of B cells in bladder cancer provided new insights into the complexity of the bladder immune system. The interferon-stimulated B-cell subtype (B-ISG15) was significantly enriched, indicating strong interferon signaling in MIBC. In this study, the main cell types and their localization characteristics within TLSs were analyzed in detail, highlighting the prognostic significance of TLSs. The dataset generated in this investigation constitutes a valuable resource that can be used to expedite the advancement of precision medicine for MIBC.

### Supplementary Information

**Additional file 1: Fig. S1.** Cellular communication between T cells and B cells in MIBC. (A) Signaling heatmaps show the roles of cell subtypes in the CXCL signaling pathway. (B) Ligand‒receptor pathway contribution diagram. (C) tSNE plot showing the expression levels of *CXCL13* in T cells (right) and *CXCR5* in B cells (left). **Fig. S2.** Validation of a prognostic value of B-cell subtypes and TLSs in MIBC. (A) Kaplan–Meier curve result of B-cell subtypes verified by the IMvigor210 dataset. (*p* < 0.05). (B) Kaplan–Meier curve result of *MS4A1 (CD20), CD3d, LMO2, AICDA* and *NEIL1* gene combination expression verified by the IMvigor210 dataset. (*p* < 0.05). **Fig. S3.** The potential significance of B-cell subtypes for prognosis in MIBC. (A) The expression levels of *LMO2, AICDA* and *NEIL1* were different between bladder cancer and normal tissues in the TCGA. (B) The Kaplan‒Meier method was used to analyze the effect of the combined expression of *MS4A1 (CD20)*, *CD3d, LMO2, AICDA and NEIL1 *gene on the survival rate of patients with MIBC (*p* < 0.05). **Fig. S4.** Schematic of the network of TLSs in MIBC. In MIBC, T cells recruit B cells through the B lymphocyte chemokine CXCL13, promote their infiltration and form B-cell regions. Surrounding these B-cell regions are various types of T cells, which collectively form T-cell regions. Additionally, DCs were found to be interspersed within the T-cell regions.

**Additional file 2: Table S1.** Patients information and sequencing statistics.

**Additional file 3: Table S2.** The key module genes.

## Data Availability

The datasets used or analyzed during the current study are available from the corresponding author on reasonable request.

## Abbreviations

ScRNA-seq: Single-cell RNA sequencing
MIBC: Muscle-invasive bladder cancer
TLSs: Tertiary lymphoid structures
TME: Tumor microenvironment
NMIBC: Non-muscle invasive bladder cancer
