# Supplementary figures and images for: Single-cell sequencing reveals the heterogeneity of B cells and tertiary lymphoid structures in muscle-invasive bladder cancer
Source: J Transl Med. 2024 Jan 12;22:48. doi: 10.1186/s12967-024-04860-1 (PMC10787393; doi:10.1186/s12967-024-04860-1)

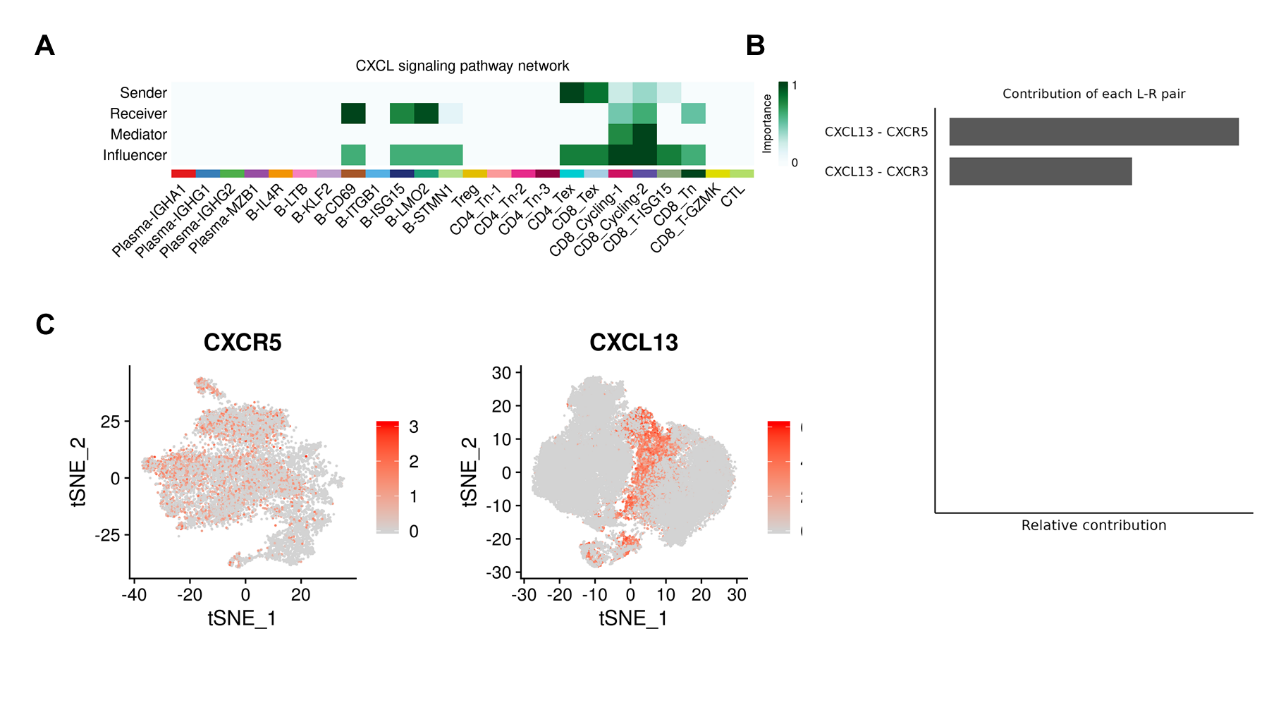


Supplementary Figure 1


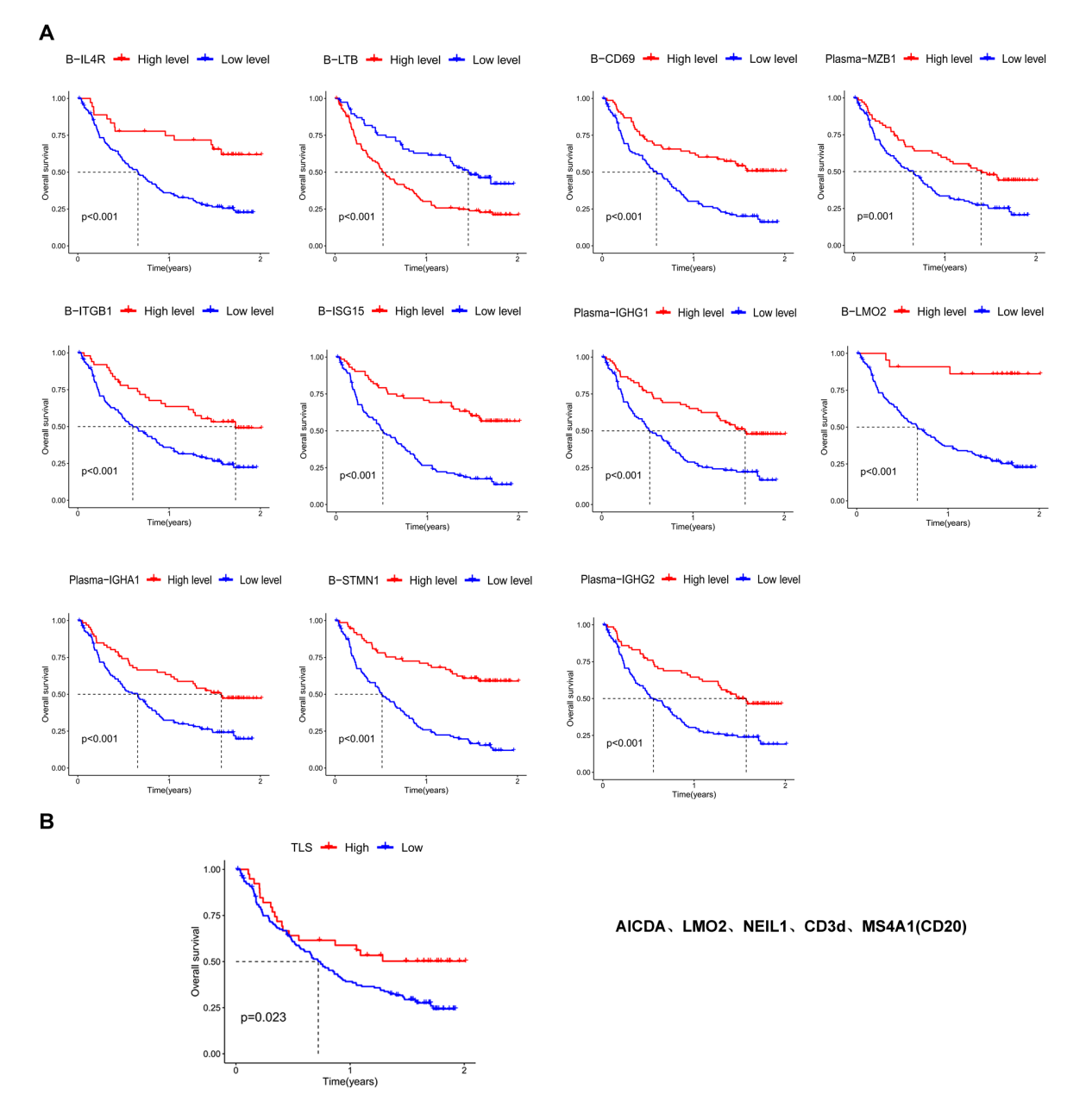


Supplementary Figure 2


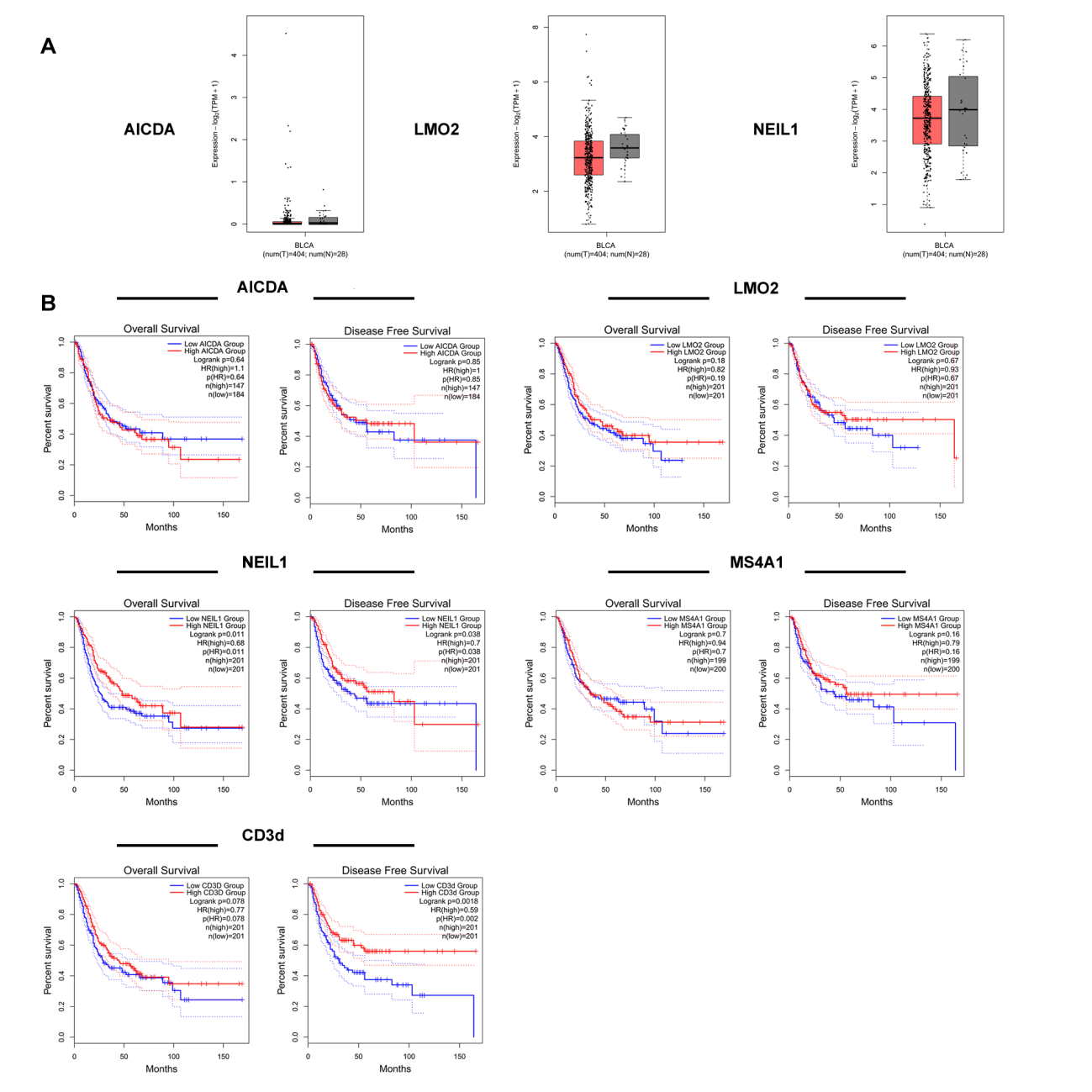


Supplementary Figure 3


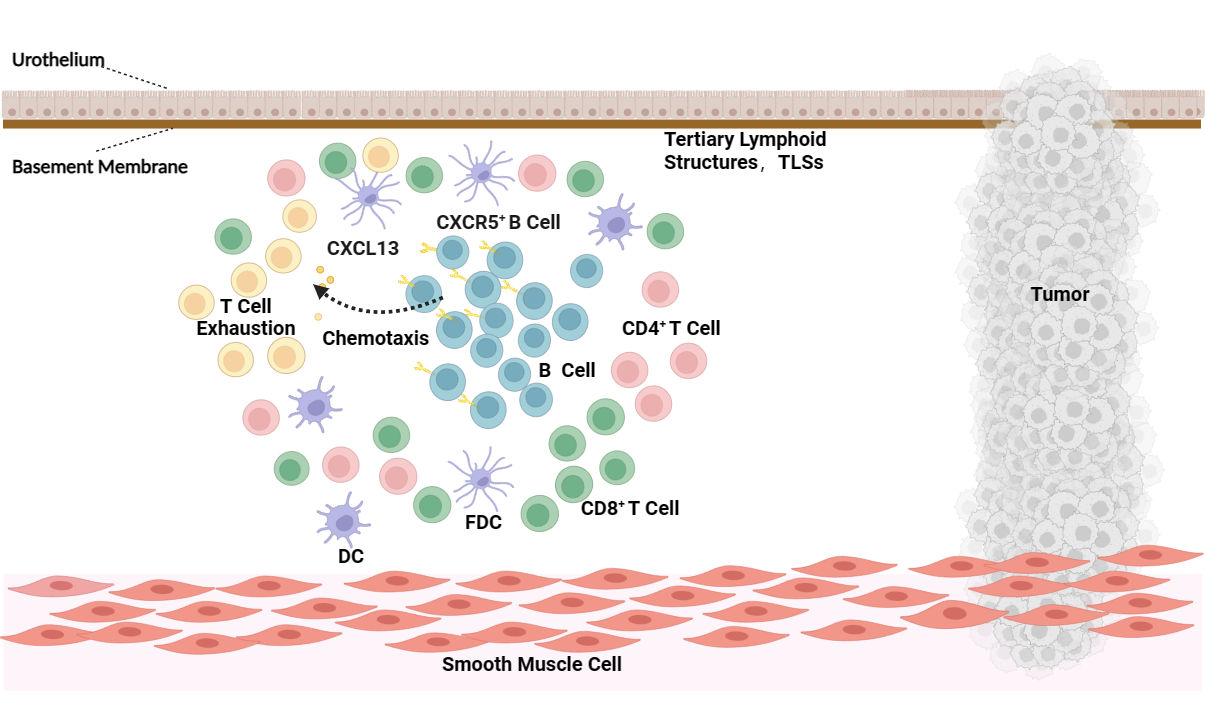


Supplementary Figure 4

Supplement: Supplementary file 1 — Additional file 1: Fig. S1. Cellular communication between T cells and B cells in MIBC. (A) Signaling heatmaps show the roles of cell subtypes in the CXCL signaling pathway. (B) Ligand‒receptor pathway contribution diagram. (C) tSNE plot showing the expression levels of CXCL13 in T cells (right) and CXCR5 in B cells (left). Fig. S2. Validation of a prognostic value of B-cell subtypes and TLSs in MIBC. (A) Kaplan–Meier curve result of B-cell subtypes verified by the IMvigor210 dataset. (p < 0.05). (B) Kaplan–Meier curve result of MS4A1 (CD20), CD3d, LMO2, AICDA and NEIL1 gene combination expression verified by the IMvigor210 dataset. (p < 0.05). Fig. S3. The potential significance of B-cell subtypes for prognosis in MIBC. (A) The expression levels of LMO2, AICDA and NEIL1 were different between bladder cancer and normal tissues in the TCGA. (B) The Kaplan‒Meier method was used to analyze the effect of the combined expression of MS4A1 (CD20), CD3d, LMO2, AICDA and NEIL1 gene on the survival rate of patients with MIBC (p < 0.05). Fig. S4. Schematic of the network of TLSs in MIBC. In MIBC, T cells recruit B cells through the B lymphocyte chemokine CXCL13, promote their infiltration and form B-cell regions. Surrounding these B-cell regions are various types of T cells, which collectively form T-cell regions. Additionally, DCs were found to be interspersed within the T-cell regions. [file 12967_2024_4860_MOESM1_ESM.docx]
